# Correction: Facile synthesis of CuS mesostructures with high photothermal conversion efficiency

**DOI:** 10.1039/d0ra90107e

**Published:** 2020-10-19

**Authors:** Lianjiang Tan, Zhongyu Wu, Xiaojing Wang, Jie Sun

**Affiliations:** Institute of Materia Medica, Shandong Academy of Medical Sciences Jinan 250062 PR China mls_sunj@ujn.edu.cn 94724500@qq.com +86-531-82919963 +86-531-67816486; Shanghai Center for Systems Biomedicine, Key Laboratory of Systems Biomedicine, Ministry of Education, Shanghai Jiao Tong University Shanghai China; School of Medicine and Life Science, University of Jinan Jinan 250200 PR China

## Abstract

Correction for ‘Facile synthesis of CuS mesostructures with high photothermal conversion efficiency’ by Lianjiang Tan *et al.*, *RSC Adv.*, 2015, **5**, 35317–35324. DOI: 10.1039/C5RA01835H.

The authors wish to correct Fig. 6c–e in the original version of this paper as it unfortunately contained incorrect images. The corrected version of Fig. 6c–e is provided below as Fig. 1. The authors confirm that these errors do not affect the scientific findings and conclusions of the paper, and sincerely apologize for the errors and any confusion.

**Fig. 1:**
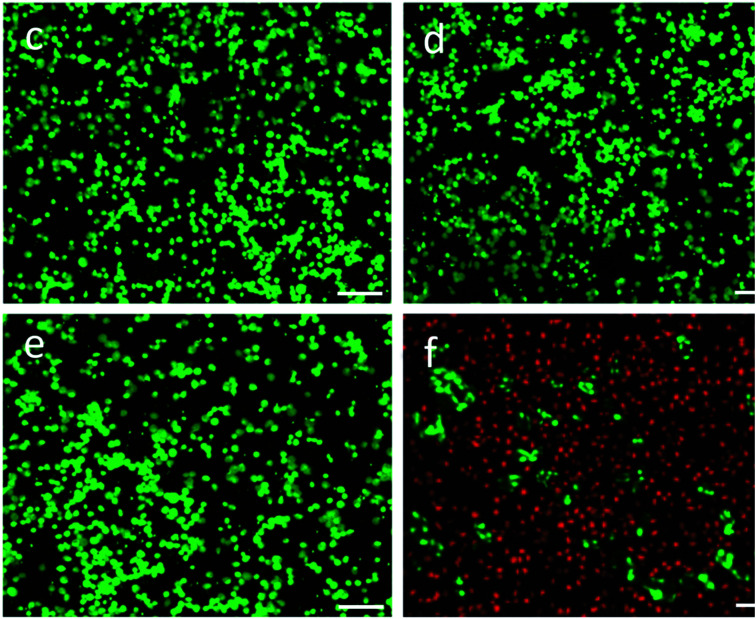
Fluorescence images of HeLa cells before and after irradiation by 980 nm laser with the power density of 0.5 W cm^−2^ over a period of 5 min in the absence (c and d) and presence (e and f) of the CuS mesostructures with the concentration of 0.3 g L^−1^. The living cells were labeled by calcein AM (green emission), and the dead cells were labeled by propidium iodide (red emission). The scale bar represents 100 mm.

## Supplementary Material

